# Inhibitor of Interleukin-1 Receptor-associated Kinases 1/4, Can Increase the Sensitivity of Breast Cancer Cells to Methotrexate

**DOI:** 10.22088/IJMCM.BUMS.8.3.200

**Published:** 2019

**Authors:** Samaneh Rahemi, Seyed Noureddin Nematollahi-Mahani, Athena Rajaie, Hossein Fallah

**Affiliations:** 1 Department of Clinical Biochemistry, Afzalipour School of Medicine, Kerman University of Medical Sciences, Kerman, Iran; 2 Department of Anatomy, Afzalipour School of Medicine, Kerman University of Medical Sciences, Kerman, Iran.

**Keywords:** Drug resistance, breast neoplasms, interleukin-1 receptor-associated kinase, methotrexate

## Abstract

Breast cancer is the most common type of cancer among women. Chemotherapy is one of the main methods of breast cancer treatment, but this method is increasingly affected due to drug resistance. One of the newly discovered factors associated with drug resistance in cancer cells is interleukin receptor-associated kinase 1 (IRAK1). The aim of this study was to investigate the relationship between IRAK1 inhibition and sensitivity to methotrexate (MTX). Effects of various concentrations of MTX and constant concentration (1μg/ml) of IRAK1/4 inhibitor was examined on MCF-7, BT-20, BT-549, MB-468 cell lines. Cell viability was examined by water soluble tetrazole -1, and cell apoptosis by flow cytometry. The expression of *IRAK1* and *BCRP* genes was also assessed by real-time PCR method. IRAK1 inhibitor decreased IC_50_ in all examined cell lines, but the most prominent effect was observed in MB-468. 72 h incubation of cell lines with IRAK inhibitor and MTX, significantly increased the annexin-V and annexin-V/7AAD positive cells, suggesting an apoptotic effect of IRAK on all examined breast cancer cell lines. RT-qPCR test results showed that the IRAK inhibitor had no effect on the expression of *BCRP* at any time. Our results showed that IRAK inhibitor can increase the chemosensitivity of breast cancer cell lines without effect on *BCRP* mRNA expression. IRAK inhibitor in combination with MTX can induce apoptosis in breast cancer cell lines.

Breast cancer (BC) is the most common malignancy among women. More than 25% of new cases of cancer among women, is BC and its prevalence is increasing in developed countries (1,2). 

Resistance to chemotherapy agents is an important barrier to the success of cancer treatment (3). ATP binding cassette (ABC) transporters are one of the most important known components involved in multidrug resistance (MDR) (4). Breast cancer resistance protein (BCRP) is one of the main ABC proteins, and a wide range of drugs are poured out by it from cells (5). Methotrexate (MTX) is a substrate of BCRP, and is used to treat several malignancies, including BC (6).

In 1863, observance of the migration of leukocytes to tumor tissues led to the discovery of the relationship between cancer and inflammation for the first time (7). Nuclear factor kappa B (NFκB) mediates inflammation pathways and is associated with apoptosis and cell migration (8). NFκB has been commonly seen in cell lines and tumor samples, and leads to drug resistance and malignancy in most forms of human cancer such as lymphoma, breast, prostate, lung, colon, pancreas, head and neck (9).

Upstream NFκB signaling pathway, interle-ukin -1 receptor- associated kinases (IRAKs) are the key intermediaries for the signaling processes from toll-like receptors (TLRs) and Interleukin-1 receptors (IL-1Rs). The function of Myeloid differentiation primary response 88 (MYD88) as the main adaptor of this pathway is to activate serine/threonine kinases of the IRAK family, and thus activate NFκB. Among the four members of the IRAK family, IRAK1 and IRAK4 are serine/threonine kinases (10).

Effective and safe NFκB clinical inhibitors are not yet available (11), but there is a specific inhibitor of IRAK1/4 as an effective suppressor of the NFκB signaling pathway, whose cancer selectivity effect was approved in studies on BC cell lines (12). This study was performed to evaluate the effect of this inhibitor on the chemo-sensitivity of BC cells to MTX. Also, because of the effects of BCRP on MTX efficacy, we evaluated the transcription level of BCRP under IRAK inhibitor treatment.

## Materials and methods

**Cell culture**

Human breast cancr cell lines MCF-7, BT-549, MDA-MB-468, and BT-20 were procured from the Iranian Biological Research Center. All cells were cultured in a humidified atmosphere of 5 % CO_2_ at 37 °C and in their own dedicated culture media. The MCF-7 cells were cultured in DMEM (Gibco, USA) medium and fetal bovine serum (FBS, Gibco,USA) 10% and 100 units/ml of penicillin, and 100 μg/ml of streptomycin. The specific culture medium of BT-549 was the same medium required by MCF-7 plus 2 mM L-glutamine. The BT-20 and MDA-MB-468 cells were cultured in Ham´s F12 (Gibco, USA) medium plus 2 mM L-glutamine and FBS 10%, and 100 units/ml of penicillin, and 100 μg/ml of streptomycin.

**Water soluble tetrazole -1 cell viability assay**

The effects of MTX (Sigma, USA), IRAK1/4 inhibitor, and combination of MTX and IRAK1/4 inhibitor (Sigma, USA) on the survival of different BC cell lines were investigated by using water-soluble tetrazole-1 (WST-I, Roche, Germany) assay. 200 µl of complete medium containing ten thousand cells with a viability of 100%, was added 24 h before the WST-1 assay to each well of the 96-well plate. After 24 h, which is necessary for cells to adhere to the bottom of the wells, the supernatant medium was discharged and replaced by 200 µl of fresh medium. Then, cells were treated with different concentrations of dissolved MTX in DMSO (100, 10, 1, 0.1, 0.01, and 0.001 μg/ml). In the IRAK1/4 inhibitor (Sigma, USA) treatment and the combined treatment of MTX and IRAK1/4 inhibitor, a volume of stock IRAK1/4 inhibitor dissolved in DMSO (Merck, Germany) was used to reach the inhibitor’s concentration in each well to 1 μg/ml. This test was repeated for all 4 cell lines. After 72 h, one-tenth of the volume of each well (20 µl) of WST-1 reagent was added to each well and the absorption was measured with ELISA reader at a wavelength of 460 nm. Each experiment was repeated tree times. The IC_50_ (half-maximal inhibitory concentration) of the medications was determined for each of the BC cell lines. For evaluation of the synergistic, additive or antagonistic effect of IRAK inhibitor on MTX, the combination index (CI) was calculated by CompuSyn software.

**Apoptosis assay by flow cytometry**

The effect of IRAK1/4 inhibitor and MTX on apoptosis was investigated in four cell lines MCF-7, BT-20, BT-549, MB-468. The cells were cultured in a 12-well plate at a density of 2×10^5 ^cells/well. After assuring the adhesion of cells to the plate, they were treated at a concentration of 1 μg/ml of MTX and in the presence of 1 μg/ml of specific IRAK inhibitor and then incubated for 72 h. The cell sample that was not treated with MTX or the inhibitor (zero concentration) was used as a negative control. After incubation, the cells were separated from the plate by trypsin-EDTA solution, centrifuged, and washed twice with PBS. The BT-20, BT-549, MB-468 cells were stained with annexin V apoptosis detector kit (BD Biosciences, USA). After emptying the supernatant PBS, 100 μl of binding buffer (1x), 5 μl of annexin V-PE conjugate, and 5 μl of 7-aminoactinomycin D (7-AAD) staining solution were added to each cell package. After a gentle vortex, the specimens were incubated for 15 min in the dark. Then, 400 μl of binding buffer (1x) was added to the cell suspension and analyzed by the Becton Dickinson FACS flow cytometer (USA). The percentage of apoptotic cells was determined using the Cell Quest software. Cells with positive annexin V and negative 7AAD were considered in the early phase of apoptosis, and cells with positive annexin V and 7AAD were considered in the late stage of apoptosis. A propidium iodide (PI) staining solution was used in the flow cytometry of the MCF-7 cells.

**Quantitative real-time PCR**

Total RNA from about three million cells of each cell line (MCF-7, BT-20, BT-549, MB-468) was extracted with the RNeasy mini kit (Qiagen, Germany) according to the manufacturer’s guidelines. The RNA concentration was determined by the ultraviolet (UV) light absorbance at 260 nm and 280 nm (ND-1000 Nanodrop). The quality of RNA was confirmed by ethidium bromide staining of 18S and 28S ribosomal RNA bands after electrophoresis in a 2% agarose gel.

The sequences of the *IRAK1 *primers which were designed using Oligo 7 software were forward, 5′- ACTGGCCCTTGGCAGCTC -3′; reverse, 5′- GGCCAGCTTCTGGACCATC-3′. The sequences of the *BCRP* primers were forward, 5′-TTCGGCTTGCAACAACTATG-3; reverse, 5′-TCCAGACACACCACGGATAA-3′. The sequen-ces of the Actin primers were forward, 5′-GACTACGAGACCGAGCTCCAGGAGT-3′; rev-erse, 5′-TGGACACCTCCGAAGTCCTTGCCC AA -3′.

The cycle of threshold (CT) value was determined for each sample. ΔCT was calculated using the equation: ΔCT= CT of ABCB1 –CT of actin. Changes of expression were calculated by the equation: 2^-ΔΔCT^.

**Statistical analysis**

All data are presented as mean ± SEM. The differences of MTX IC50 and gene expression were analyzed by student t-test. IC50 was calculated using Probit regression analysis. Satistical analyses were performed using the SPSS software version 20 for Windows. P<0.05 was considered as statistically significant.

## Results

**IRAK inhibitor effects on IC**_50_ **values**

**Fig. 1:**
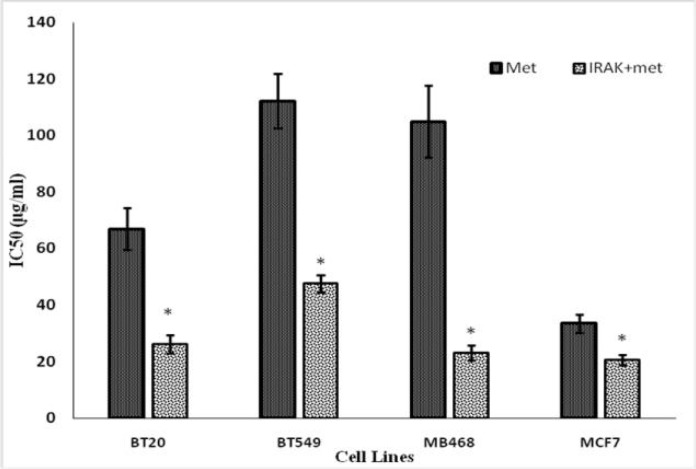
The effect of IRAK inhibitor on the reduction of IC_50_ of Methotrexate in MCF-7, BT20, MB468 and BT549 cell lines**. The cells were exposed to different drug concentrations (0.001, 0.01, 0.1, 1, and 10 μg/ml) in the presence of a constant amount of IRAK inhibitor (1 μg/ml) for 72 h. Then the apoptosis rate was measured by WST1 kit and IC**_50_** was calculated by Probit regression test**

As figure 1 shows, the analysis of the WST-1 test results indicated that in MCF-7 cell line, the IC50 values for MTX (1 μg/ml) at 72 h treatment was 33 μg/ml. These values, as a result of the treatment of the cells by adding IRAK inhibitor (1 μg/ml) to the culture medium at similar times and conditions, led to a reduction of IC50 to 20 μg/ml of the culture medium (P =0.043).

IC50 values in BT-20 cell line for MTX (1 μg/ml) were 67 μg/ml of the culture medium, where treatment of cells with IRAK inhibitor (1 μg/ml) reduced this value to 26 for MTX (figure 1) (P<0.0001).

**Fig 2:**
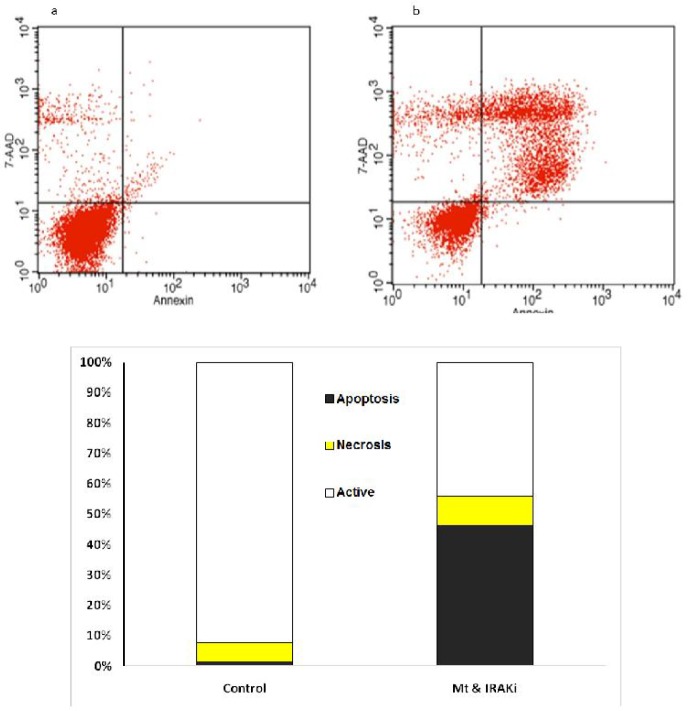
Comparison of the percentage of living cells, necrosis, and apoptosis in the BT-20 cell line. **a:** **Control; b: IRAK inhibitor** **+** **MTX; c: presenting chart; The comparison was performed in the control group treated with MTX (1 μg/ml) and IRAK inhibitor (1 μg/ml) for 72 h**

**Fig 3:**
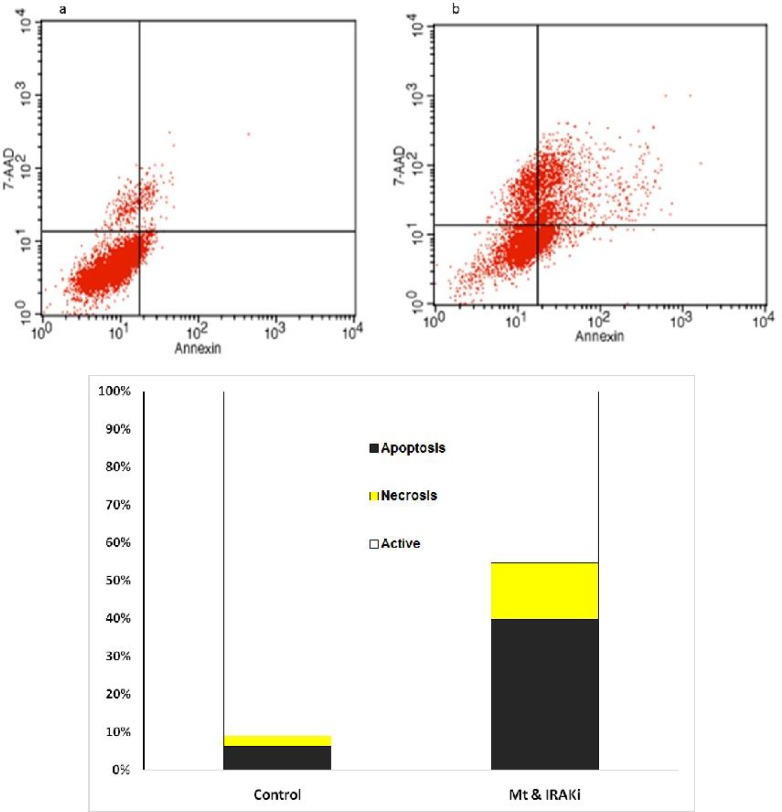
Comparison of the percentage of living cells, necrosis, and apoptosis in the BT-549 cell line. a**: control; b: IRAK inhibitor+MTX; c: presenting chart; The comparison was performed in the control group treated with MTX (1 μg/ml) and IRAK inhibitor (1 μg/ml) for 72 h**

In the BT-549 cell line, IRAK inhibitor (1 g/ml) decreased the IC_50_ of MTX (1 μg/ml) from 112 to 48 μg/ml of the culture medium (P <0.0001).

Finally, in the MB-468 BC cell line, the IC_50_ value of MTX (1 μg/ml) was 105 μg/ml of the culture medium. The treatment of cells in the same conditions with the IRAK inhibitor (1 μg/ml) recorded an IC_50_ value of 23 μg/ml for MTX (P<0.0001).

Combination Index for MCF7, MB468, BT549 and BT20 cell lines was 0.964, 0.168, 0.241 and 0.272, respectively. With the exception of the MCF7 cell line, IRAK inhibitor in other cells showed an obvious synergic effect on MTX.

**IRAK inhibitor effects on apoptosis of BC cell lines**

**Fig 4:**
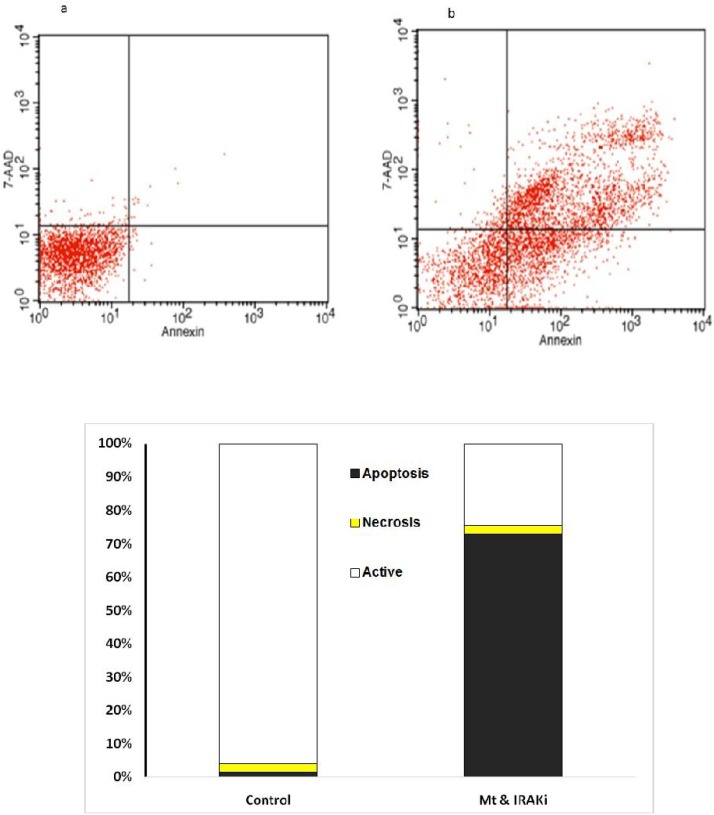
Comparison of the percentage of living cells, necrosis, and apoptosis in MB-468 cell line. **a: control; b: IRAK inhibitor+MTX; c: presenting chart; The comparison was performed in the control group treated with MTX (1 μg/ml) and IRAK inhibitor (1 μg/ml) for 72 h**

**Fig 5:**
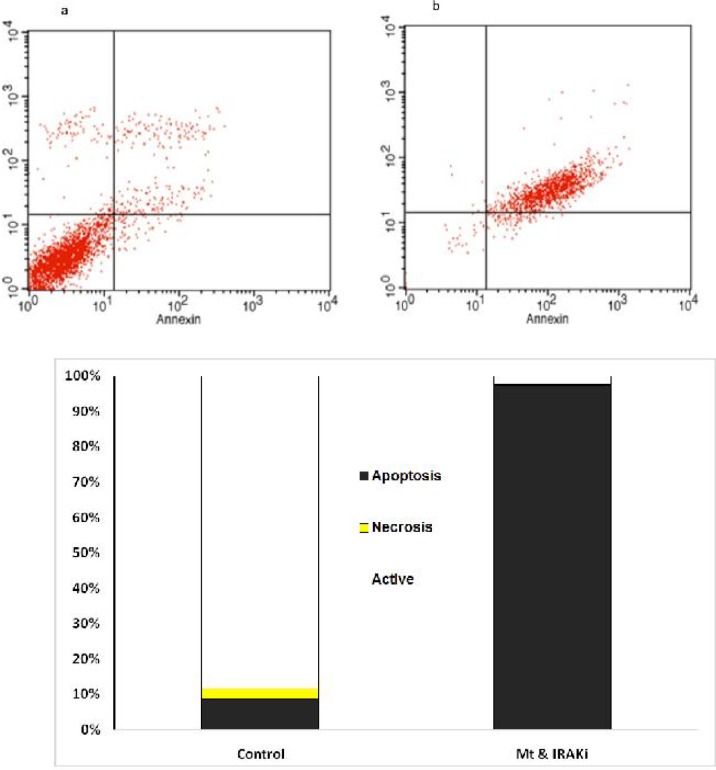
Comparison of the percentage of living cells, necrosis, and apoptosis in MCF-7 cell line **a: control; b: IRAK inhibitor+MTX; c: presenting chart; The comparison was performed in the control group treated with MTX (1 μg/ml) and IRAK inhibitor (1 μg/ml) for 72 h**

The effects of IRAK inhibitor on apoptosis was assessed on BC cell lines. As figures 2 to 5 show, 72 h incubation of BT-20, BT-549, MB- 468, and MCF-7 cell lines with IRAK inhibitor and MTX significantly increased the annexin-V and annexin-V/7AAD positive cells, suggesting an apoptotic effect of IRAK on all tested BC cell lines.

**Effect of IRAK inhibitor on the transcription of** ***IRAK*** **and** ***BCRP*** **genes**

**Fig 6:**
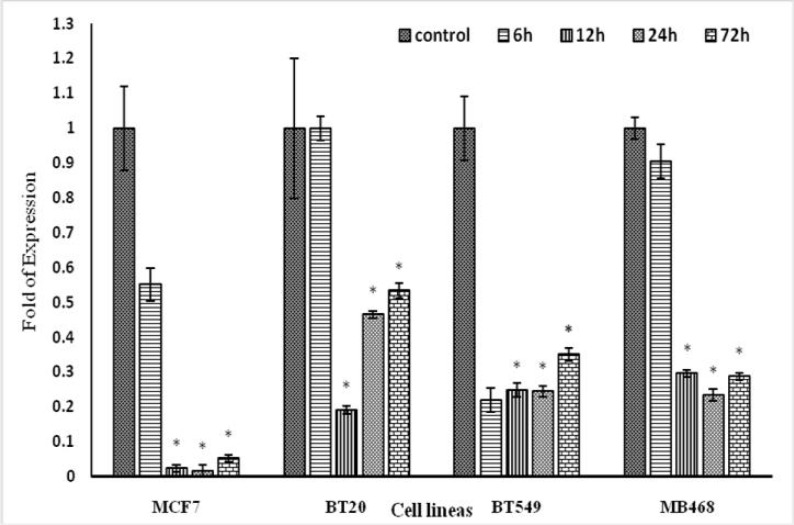
**Effects of IRAKi on** ***IRAK.1*** **gene transcription in BC cell lines. **The cells were treated with IRAK1/4 inhibitor (1 μg/ml) for 6, 12, 24 or 72 h,, then total RNA was extracted and mRNA level of *IRAK* was assayed by real-time PCR in comparison with control group (without any IRAK inhibitor). Results are reported in three replicates (P<0.05*).

**Fig 7:**
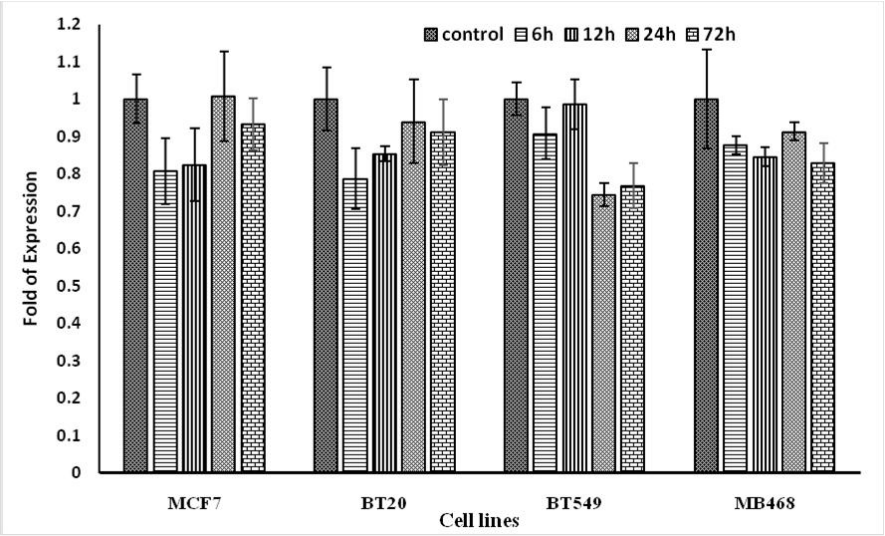
**Effects of IRAKi on** ***BCRP*** **gene transcription in BC cell lines.**The cells were treated with IRAK1/4 inhibitor (1 μg/ml) for 6, 12, 24 or 72 h, then total RNA was extracted and mRNA level of *BCRP *was assayed by real-time PCR in comparison with control group (without any IRAK inhibitor). Results are reported in three replicates (P < 0.05*).

Figures 6 and 7 show the effect of IRAK inhibitor on the expression of *IRAK* and *BCRP* in four BC cell lines. IRAK inhibitor reduced the expression of *IRAK* gene at 12, 24, and 72 h,but it had no effect on the expression of *BCRP *gene.

## Discussion

Chemotherapy is the most effective treatment for advanced cancers (13). Contrary to significant advances, most drug regimens used to prevent irreversible damage of the disease ultimately fail to control the progression of the disease, the most important reason of which is the spread of drug resistance in tumor cells between treatment cycles (14). Drug resistance is responsible for 90% of chemotherapy failures (15).

Resistance to chemotherapy agents is a set of phenomena that occur based on changes in apoptosis, cell cycle, and drug metabolism (16). It appears that the effective factor in drug failure is drug resistance created by ABC transporters (17).

MTX is a substrate for the BCRP pump (18). Also, Hanz et al. showed that different concentrations of MTX increased the expression level of the ABCB1 protein (19). Increased expression of BCRP is reported in many cancers which protects cancer cells against MTX and topotecan drugs (20).

In the present study, WST-1 test results for the first time showed that the concomitant use of IRAK and MTX reduced the IC_50_ of these drugs in all BT-20, BT-549, MB-468, and MCF-7 cell lines. The lowest inhibitory effect of IRAK1 on the MTX sensitivity elevation was observed in MCF-7 cells. Due to the high expression of IRAK in triple negative BC cells in comparison with other types of BC cells (12), the reduction of IC_50_ in BT-20, BT-549 and MB-468 cell lines, that are triple negative BC, is more than that of MCF-7 cell line, which is not in this group of BC cells.

The greatest effect of IRAKi in reducing the IC_50_ of MTX was in the MB- 468 cell line. A comparison of expression of *IRAK1* in metastatic clinical tumor samples showed that the expression and activity of IRAK1 increased when the tumor was metastatic, thus became more sensitive to IRAK1 inhibition (12). This could justify the highest reduction of IC_50_ for MTX as a combination with IRAK inhibitor in the MB-468 cell line.

IL-1β released by tumor cells increases the expression of IL-1 and increases the level of phosphorylated IRAK1, which leads to the activation of NF-κB and the stimulation of important genes for the growth and spread of tumors such as *IL-6*, cyclooxygenase 2 (*COX-2*), brain-derived neurotrophic factor(*BDNF*), and (interferon regulatory factor-2 (*IRF-2*)(21). *NF-κB* inhibition appears to be able to decrease drug resistance, but the use of NF-κB inhibitors may have unwanted effects on different signaling deriving from it (22).

Similarly, inhibition of P-38 can induce apoptosis in cells resistant to paclitaxel, and both IRAK1 and P-38 MAPK inhibitors decrease the expression of the anti-apoptotic myeloid cell leukemia 1 (MCL-1) protein(12).

Therefore, the activation of IRAK1 appears to play an important and crucial role in causing acquired chemotherapy resistance (12). Cheng et al. showed that inhibiting IRAK1 makes hepatocellular carcinoma cells sensitive to doxorubicin and Sorafenib by suppressing apoptotic cascade *in vitro* (23). Many cancers that show elevated levels of IRAK1 and IRAK4 are resistant to chemotherapy, which highlights the role of IRAK in the development of drug resistance (24). 

Flow cytometry using annexin-V and propidium or 7AAD showed increased induction of apoptosis in BC cell lines by concomitant use of IRAK inhibitor and MTX.

A survey conducted by Boukerche et al. on metastatic human melanoma cell lines showed that *IRAK1* is one of the 8 genes expressed in metastatic cells in comparison with parental human melanoma cell lines (25).

A study by Wee et al. on BC cell line showed that there is an 80% increase of *IRAK* expression in BC cells, and that increased expression of *IRAK1* played a role in creating resistance to paclitaxel (12). Since MTX is a substrate of BCRP (18), it is likely that one of the IRAK inhibiting mechanisms in creating drug resistance is by increasing the ABC transporters.

IL-1β induces NF-κB activity and expression of *BCRP *in some types of BC and normal cell lines (26). A study by Gao et al. showed that cellular stress, such as DNA damage and different activation signals like NF-κB, can induce the expression of multidrug resistance 1 (*MDR-1*) gene and its activation (27). DeGraffenried et al. showed that there is an increase in NF-κB activity in the tamoxifen-resistant BC cell lines, but the study did not report a relationship between the high activity of NF-κB and ABC transporters (28).

Clearly, both groups of cytokines induced by NF-κB and the P-38 signaling pathways play a role in resistance to paclitaxel. Drug-targeting of IRAK-1 may be an effective treatment option for advanced metastatic triple negative BC because it is enough for blocking both NF-κB and P-38 signaling (12). 

The RT-qPCR results of this study showed that the IRAK inhibitor has no effect on the expression of *BCRP*, and therefore the effect of IRAK inhibitor on increased apoptotic sensitivity of BC cell lines, may be due to different regulatory signaling pathways.

A study by Zhang et al. showed that increased expression of *BCRP *in MCF-7/HER2 cells was through PI3K/AKT signaling pathway and NF-κB activation (29). Porcelli et al. showed that EGFR and PKB (AKT) pathways play a role in regulating *BCRP* expression in lung cancer cell lines. Wang et al. suggested that P53 wild-type acts as a negative transcription factor for the *BCRP* gene (3). Activation of the PI3K/AKT/mTOR signaling pathway is an important mechanism for tamoxifen resistance (30).

Most chemotherapy drugs have a low therapeutic efficacy, and rarely distinguish between normal and malignant cells, a problem often reinforced by the onset of inevitable resistance and relapse of the disease. Regarding the wide range of chemotherapy drugs that are substrates of BCRP, it appears that finding an effective drug strategy to overcome drug resistance with a selectivity property of cancer cells to reduce drug resistance to chemotherapy agents can be a hope for more successful treatment of the patients, which requires the exploration of other signaling pathways affecting MTX drug resistance and BCRP transporter.
